# Meteorological Report for November

**Published:** 1880-12

**Authors:** 


### METEOROLOGICAL REPORT FOR OCTOBER, 1880.

| RAIN. DATE | IN. | Mean daily Barometer. | Mean daily Thermometer | Mean daily Humidity. | Maximum Thermometer | Minimum Thermometer | Prevailing direction of Wind. | Maximum hourly velocity of Wind, (Daily.) |
|---|---|---|---|---|---|---|---|---|
| 1 | 0 | 30.138 | 54.0 | 45.7 | 61 | 40 | E. | ......... |
| 2 | 0 | 30.133 | 53.2 | 53.0 | 60 | 47 | E. | ......... |
| 3 | 22 | 30.088 | 49.2 | 85.3 | 54 | 46 | E. | ......... |
| 4 | 41 | 30.041 | 57.5 | 89 0 | 61 | 49 | E. | ......... |
| 5 | 12 | 30.026 | 61.2 | 93.7 | 64 | 57 | E. | ......... |
| 6 | 36 | 29.914 | 53.5 | 76.7 | 68 | 44 | N. W. | ......... |
| 7 | 0 | 30 124 | 44.0 | 45.7 | 51 | 36 | N. W. | ......... |
| 8 | 0 | 30 089 | 49 2 | 48.0 | 55 | 36 | E | ......... |
| 9 | 0 | 30 119 | 53 5 | 63.7 | 61 | 40 | E. | ......... |
| 10 | 2 19 | 29.957 | 63.5 | 84 7 | 66 | 52 | S. E. | ......... |
| 11 | 0 | 30 175 | 56.5 | 54.0 | 65 | 50 | N. W. | ......... |
| 12 | 0 | 30.311 | 57.2 | 47.3 | 65 | 45 | N. W. | ......... |
| 13 | 02 | 30.280 | 53.2 | 63.0 | 61 | 47 | E. | ......... |
| 14 | 45 | 30.126 | 45.7 | 71 3 | 53 | 43 | N. W. | ......... |
| 15 | 0 | 29.985 | 46 0 | 42 3 | 50 | 35 | N. W. | ......... |
| 16 | 0 | 30.183 | 51.2 | 47.7 | 55 | 40 | S. W. | ......... |
| 17 | 0 | 30.202 | 50.2 | 52.0 | 55 | 46 | E. | ......... |
| 18 | 08 | 30.255 | 38 2 | 70.0 | 50 | 31 | N. W. | ......... |
| 19 | 0 | 30.124 | 41.2 | 60.3 | 51 | 26 | E. | ......... |
| 20 | 0 | 30.108 | 42.0 | 58.7 | 52 | 35 | N. W. | ......... |
| 21 | 0 | 30.439 | 37.7 | 43.3 | 47 | 28 | N. E. | ......... |
| 22 | 0 | 30.482 | 37.7 | 33.7 | 45 | 30 | N. | ......... |
| 23 | 0 | 30.561 | 34.5 | 41.0 | 43 | 25 | E. | ......... |
| 24 | 01 | 30.472 | 31.5 | 57.0 | 37 | 25 | E. | ......... |
| 25 | 41 | 30.212 | 40.2 | 85.3 | 48 | 30 | N. E. | ......... |
| 26 | 16 | 30.239 | 46.7 | 84.3 | 50 | 41 | Calm. | ......... |
| 27 | 49 | 30.277 | 43 2 | 94 7 | 48 | 42 | E. | ......... |
| 28 | 1.18 | 30.090 | 50.0 | 95.3 | 57 | 40 | E. | ......... |
| 29 | 24 | 30.166 | 53.5 | 80 0 | 61 | 47 | N. W. | ......... |
| 30 | 1.87 | 30.076 | 37.7 | 87.7 | 51 | 41 | E. | ......... |
| | | ........ | ...... | ...... | ... | ... | ......... | ......... |
| Sums | | 30 180 | 48 1 | 65.1 | 54.8 | 39 8 | ......... | ......... |

Highest Barometer, 30.603, on the 23rd, 7:00 A.M.
Lowest Barometer, 29.733, on the 6th, 10:31 A.M.
Monthly range of Barometer, 0.870.
Highest Temp. 68°, on the 6th. Lowest Temp. 25°, on the 23d and 24th.
Monthly range of Temperature, 43°.
Greatest daily range of Temperature, 25°, on the 19th.
Least daily range of Temperature, 6°, on the 27th.
Mean of Maximum Temperatures, 54°.8.
Mean of Minimum Temperatures, 39°.8.
Total rainfall or melted snow, 8 21 inches.
Prevailing wind, East. Total movement of wind, 57.57 miles.
Maximum velocity of wind and direction, 25 miles per hour; S.W., on 6th.

H. HALL, Corp'l. Signal Service U.S. A

Atlanta, Ga., November, 1880.

